# Translating behavioral interventions into virtual reality: the *Transcend Framework* for immersive health design

**DOI:** 10.3389/fdgth.2026.1766741

**Published:** 2026-03-31

**Authors:** Rosalba Hernandez, Killivalavan Solai, Soonhyung Kwon, Prasakthi Venkatesan, Drew Fast, Sandraluz Lara-Cinisomo, Harris J. Nisar

**Affiliations:** 1 School of Social Work, University of Illinois at Urbana-Champaign, Urbana, IL, United States; 2 Center for Innovation in Teaching & Learning, University of Illinois at Urbana-Champaign, Urbana, IL, United States; 3 School of Social Work, College of Behavioral & Community Sciences, University of South Florida, Tampa, FL, United States; 4 Department of Health and Kinesiology, College of Applied Health Sciences, University of Illinois Urbana-Champaign, Champaign, IL, United States; 5 Health Care Engineering Systems Center, College of Engineering, University of Illinois at Urbana-Champaign, Urbana, IL, United States

**Keywords:** chronic disease management, healthcare innovation, immersive interventions, positive psychology, user-centered design, virtual reality, digital health

## Abstract

Virtual Reality (VR) has evolved from entertainment to a versatile platform for clinical and public health innovation. In medicine, VR supports pain management, rehabilitation, and cognitive training, and shows growing promise for addressing chronic diseases linked to modifiable risk factors. To support this expansion, we introduce the *Transcend Framework*, the Translational Engineering of Behavioral Interventions into Immersive Contexts for Engagement and Design framework, a systematic model for adapting evidence-based behavioral interventions into VR platforms, illustrated by *Joviality™*, a positive psychological intervention designed for use during hemodialysis. The aim of this paper is to outline a clear, reproducible process for translating behavioral interventions into immersive digital formats that supports broader research, clinical, and implementation applications. The framework comprises five stages: (1) identifying the target population; (2) assessing feasibility and adapting the curriculum for VR; (3) pre-production planning, including storyboarding and design specification; (4) previsualization and asset creation of immersive environments; and (5) iterative VR development and testing to refine usability, accessibility, and engagement. Each stage emphasizes user-centered design and attention to physical limitations, cognitive load, and accessibility to ensure feasibility and effectiveness. Interactive, visually rich, modular environments foster engagement, while gamified activities enhance experiential learning and skill acquisition, and culturally attuned content ensures inclusivity. Continuous, data-informed refinement guided by end-user feedback ensures usability and sustained engagement. This methodological framework provides a practical roadmap for developing and optimizing VR-based behavioral health interventions and demonstrates how immersive technology can advance health education, promote behavior change, and enable scalable, equitable implementation across clinical contexts.

## 1 Introduction

Virtual Reality (VR) technology has evolved substantially since its commercial debut in the early 1990s (1). Initially developed for gaming, VR quickly enabled immersive exploration and interactive play (2), exemplified by early innovations such as *Dactyl Nightmare* (1991), one of the first multiplayer experiences to demonstrate the medium's potential for virtual presence and shared digital environments. In this early system, players wore bulky headsets connected by thick cables to large computers as they entered a pixelated arena to battle flying dinosaurs, an experience that underscored both the promise and the technical limitations of the technology at the time. As the field matured, experiences became more sophisticated, from rhythm-based games such as *Beat Saber* (2018) to realistic roller coaster simulations, fitness challenges, and interactive escape room scenarios (3). These advances have propelled VR beyond entertainment, demonstrating its promise as a tool for simulation, education, and clinical training (3–5).

Today, VR stands at a pivotal intersection of technology and health, where immersive environments can enhance experiential learning, patient engagement, and therapeutic outcomes (4). Improvements in computing power, display resolution, and multisensory feedback have narrowed the gap between virtual and physical experience, allowing VR to emerge as a credible platform for evidence-based behavioral and clinical interventions (6). Recent applications include simulation-based training for clinicians, mental health therapies, pain management, and avatarguided programs such as *Joviality*™, a fully immersive positive psychological intervention for patients undergoing hemodialysis (7, 8).

Yet even as VR capabilities expand, translating established behavioral interventions into immersive environments remains a critical challenge. Questions persist about how to preserve theoretical fidelity, select core curriculum components, and guide digital adaptation in ways that enhance rather than dilute intervention impact. Despite this momentum, the field lacks a clear, reproducible framework to guide the translation of validated behavioral interventions into immersive VR formats. The objective of this paper is to address this gap by introducing the *Transcend Framework* (Translational Engineering of Behavioral Interventions into Immersive Contexts for Engagement and Design), a structured model that integrates behavioral science, clinical insight, and engineering design. The aim is to provide a systematic process for adapting evidence-based behavioral interventions into virtual environments while preserving theoretical fidelity, optimizing usability, and supporting implementation in diverse settings, using our *Joviality™* software as an illustrative case example.

## 2 Background

The evolution of VR hardware and software parallels advances in imaging, computing, and sensory integration, yielding systems with high-definition stereoscopic displays, spatial audio, and six-degree-of-freedom tracking, including three axes and three rotations, that minimize latency and enhance depth perception (9). These refinements have transformed VR from a novelty into a fundamental scientific and clinical tool. Its defining features of controlled immersion, repeatability, and emotional engagement make it uniquely suited to study and influence human perception, cognition, and behavior in real time (10). Within medicine and public health, VR now bridges engineering, behavioral science, and clinical practice, enabling experiential learning for clinicians, behavioral therapy for patients, and scalable interventions once limited by physical or didactic constraints (5).

At a design level, VR enables exploration of both hyper-realistic and imaginative 3D environments (11). End users engage with these digital worlds through increasingly intuitive hardware interfaces, while advanced rendering engines and real-time software architectures generate responsive virtual environments that continuously adjust to movement and gaze. Haptic feedback and advanced hand-tracking allow users to grasp and manipulate virtual objects directly, sensing vibration, resistance, and texture within the simulated environment (12). The resulting sense of presence activates multiple sensory systems in concert, producing perceptual realism that can shape cognition, emotion, and behavior (13). These capabilities now encompass applications across surgical training (14), rehabilitation, and psychosocial therapy, each enabling the controlled reproduction of complex perceptual and emotional states (11). By integrating sensory immersion with behavioral engagement, VR has become a versatile platform for experiential learning and structured skills training, underscoring its expanding role in healthcare and educational settings.

The expansion of VR into healthcare also underscores a broader imperative to deliver equitable, scalable, and patient-centered care. Traditional educational and behavioral models depend on human instructors and in-person delivery, limiting reach and consistency. Immersive digital platforms, in contrast, can extend evidence-based content to geographically remote, economically diverse, and mobility-limited populations (5). These platforms also permit the seamless integration of multilingual features within the interface, narration, and avatar interaction (15, 16). This democratization is particularly salient in chronic disease management and mental health care, where disparities in access to psychosocial interventions remain pervasive (17). Yet, as with any emerging technology, digital inequalities, including gaps in affordability, access, and digital literacy, mirror broader social determinants of health and must be addressed to ensure that these tools yield equitable impact (18, 19).

Beyond accessibility, VR's scientific relevance lies in its capacity to evoke authentic psychophysiological responses under controlled conditions. Immersive simulations can modulate autonomic activity, neural engagement, and affective states in ways that closely mirror real-world experience (20). These properties make VR not only a delivery mechanism for clinical interventions but also an experimental platform to probe the mechanisms of stress regulation, emotion processing, and learning (21). This dual function as therapeutic and investigative tool positions VR as a bridge between behavioral science and medicine, advancing the study of mind– body processes in cardiovascular, neurological, and mental health. To support this emerging field, the *Transcend Framework* provides a structured approach for translating evidence-based behavioral interventions into immersive, user-centered VR experiences.

Recent advances in augmented reality (AR), exemplified by Apple's Vision Pro, further blur the distinction between digital and physical space by superimposing virtual elements onto real-world contexts. Ongoing improvements in resolution, processing power, and haptic precision have strengthened performance and end-user endurance, reducing perceptual fatigue and allowing seamless toggling between virtual and augmented environments, such as moving from a full operating suite to an instructional setting displaying only the surgical table. These technological gains, together with a widening range of headsets from entry-level models near $400 to enterprise systems exceeding $3,000, are expanding access and accelerating adoption across clinical, educational, and research settings (22).

Health systems and academic institutions are increasingly piloting VR/AR technologies within digital-health initiatives that include telemedicine, wearables, and artificial intelligence (23). Though full enterprise deployment remains limited, there is a discernible shift from isolated proofs-of-concept toward coordinated ecosystems linking training, clinical care and research (24). Institutional adoption will depend on cross-disciplinary collaboration, rigorous evaluation and data standards that assure reproducibility and scalability. The following sections summarize empirical evidence on efficacy in medical education and patient care and introduce the *Joviality™* case study as a model for translating behavioral interventions into immersive, patient-centered experiences.

### 2.1 Virtual reality in medical education and clinical training

Empirical evidence supporting the efficacy of VR in medical education and training continues to grow, informed by both foundational studies and an expanding body of recent randomized controlled trials (RCTs) and meta-analyses. In a pivotal 2002 trial, Seymour and colleagues evaluated a computer-based virtual simulator for laparoscopic training (25). Compared with conventionally trained residents, those who trained on the simulator completed gallbladder dissection 29% faster, committed six times fewer errors, and were markedly less likely to cause gallbladder injury or burn nontarget tissue. This early work laid the foundation for contemporary immersive VR. A 2024 systematic review and meta-analysis examined the use of virtual reality in robotic surgical training. Across 18 randomized trials (*n* ≈ 339–357 across pooled outcomes), VR training significantly improved objective performance scores (SMD, 1.04; 95% CI, 0.40–1.69) and reduced task completion times compared with no additional training, while performing comparably to dry-lab simulation (26). This meta-analysis sets the stage for recent trials assessing VR's impact across other areas of medical and interprofessional training.

In an open, parallel-group randomized trial of operating-room nurses (*n* = 56) undergoing thoracic-surgery training, participants assigned to the VR intervention achieved significantly higher technical performance scores on standardized skill assessments (median 520 vs. 440; *p* = 0.04) compared with those receiving conventional instruction, and reported high user satisfaction, supporting the feasibility of VR-based training for perioperative teams (27). Evidence of VR's efficacy has also emerged in orthopedic training. In orthopedic residency training for balloon kyphoplasty [a minimally invasive procedure to restore vertebral height and stabilize compression fractures using an inflatable balloon and polymethylmethacrylate (PMMA) cement] residents who completed VR-based simulation achieved significantly higher intraoperative performance ratings on a standardized procedural checklist compared with those trained by traditional instruction (mean score increase from 2.85 ± 0.65 to 4.09 ± 0.62; *p* < 0.05), along with progressive reductions in operative time as practice increased (28).

Clinical knowledge acquisition and decision-making performance are also showing measurable gains with VR-based training tools. In a randomized crossover trial of 72 senior medical students, VR-based training for managing acute medical emergencies such as myocardial infarction produced comparable immediate post-training knowledge scores to video-based seminars but significantly greater 30-day retention (75.4% vs. 69.0%; *p* < 0.05) (29). Despite higher physiologic stress responses during simulation, participants rated the VR experience as more engaging, effective, and conducive to long-term learning (29). In cardiac point-of-care ultrasound (POCUS) training for novice learners, self-directed VR instruction was noninferior to physicianled teaching at one month, with participants in the VR group demonstrating better retention of hands-on image acquisition and interpretation skills during follow-up assessments (30). Finally, VR-based approaches have also demonstrated benefit in cardiopulmonary resuscitation (CPR) training. In a meta-analysis of nine randomized controlled trials encompassing 855 participants, including medical and nursing students as well as lay trainees, VR/AR-based CPR training achieved chest-compression depth, rate, and overall performance equivalent to that attained with conventional face-to-face instruction (31). A subsequent randomized trial of 205 participants found that although traditional instruction yielded higher immediate post-course scores, VR training achieved comparable performance by three months with improving compression quality over time, underscoring its scalability and durable efficacy (31).

Taken together, contemporary evidence indicates that VR-based instruction effectively transfers technical skills to clinical practice, performs as well as or better than conventional training in procedural disciplines, enhances teamwork and communication, and delivers CPR proficiency comparable to face-to-face instruction while enabling scalable, repeatable learning. Its pedagogic foundation lies in standardized, feedback-informed experiential training that reinforces retention, consistency, and safe application in clinical care.

### 2.2 Therapeutic efficacy and clinical applications of virtual reality

Beyond clinical training, VR has become increasingly integrated into patient care. Therapeutic applications now encompass pain management, physical rehabilitation, exposure therapy for phobias, cognitive training for dementia and attention disorders, and pre-surgical planning (3, 32–34). A pivotal milestone was achieved in 2017 with the regulatory approval of RelieVRx, the first prescription-based VR platform for chronic pain management (35). Integrating cognitive behavioral therapy with guided breathing, RelieVRx resulted in a 40% reduction in pain intensity and a 50% reduction in pain interference, outcomes that significantly exceeded those observed with a sham VR control (35–37). This milestone established VR as an evidence-based therapeutic platform capable of producing measurable and durable clinical benefits (3, 32).

Evidence for VR-based therapy continues to strengthen in the areas of pain management and physical rehabilitation. In a meta-analysis of 28 randomized trials of patients with back and neck pain (*n* = 1,114), both immersive and non-immersive VR programs incorporating interactive 3D exercises and real-time visual feedback significantly reduced pain (short-term SMD −1.79; 95% CI, −2.72 to −0.87) and improved disability scores (SMD −0.44; 95% CI, −0.72 to −0.16) compared with conventional physiotherapy-based exercise training (38). Similarly, a metaanalysis of 20 trials in chronic low-back pain (*n* = 1,059) found that immersive VR-based exercise training produced greater short-term reductions in pain (µ_difference_ −1.43; 95% CI, −1.86 to −1.00) than conventional physiotherapy, though gains in pain, fear of movement, and disability were not sustained beyond three months (39). Finally, a 2025 meta-analytic review found that immersive VR programs incorporating psycho-cognitive elements (such as pain-neuroscience education and cognitive-behavioral strategies) produced significantly greater reductions in pain-catastrophizing than exercise-only VR or non-immersive/sham control interventions (SMD ≈ −0.32; 95% CI, −0.56 to −0.09) (40). Together, these findings position VR as a credible adjunct to conventional rehabilitation, linking physical recovery with the psychological dimensions of pain and setting the stage for its use in mental health care. At the same time, although many of these programs incorporate psycho-cognitive components, immersive physical rehabilitation and motor-training interventions rely more heavily on motion tracking, graded motor demands, and sensory feedback than the curriculum-based behavioral and cognitive interventions that are the focus of this paper (41).

Moving into the mental-health arena, VR is gaining traction. In a meta-analysis of 29 studies inclusive of 1,561 stroke survivors, VR-based rehabilitation significantly reduced anxiety (SMD −0.97; 95% CI, −1.84 to −0.09) and depressive symptoms (SMD −0.94; 95% CI, −1.46 to −0.42) and improved quality of life (SMD 0.94; 95% CI, 0.42 to 1.45) compared with standard rehabilitation, with longer VR exposure (>6 weeks) producing the strongest effects, particularly in European cohorts (42). In line with these findings, a meta-analysis of 11 randomized trials involving 752 stroke patients found that immersive and non-immersive VR programs, typically gamified motor rehabilitation exercises using head-mounted displays or screen-based systems, significantly improved depressive symptoms compared with conventional physiotherapy (SMD −0.75; 95% CI, −1.35 to −0.15) (43). In social anxiety disorder, a systematic review of 18 studies found that immersive VR exposure therapy was feasible (attrition rate of 11.36%), acceptable, and effective in reducing social anxiety symptoms, though direct comparisons with traditional exposure remain limited (44). Across the mental health spectrum, studies demonstrate that VR can reduce symptoms of anxiety, depression, and post-traumatic stress, improve emotional regulation and cognitive functioning, and enhance therapeutic engagement in conditions such as anxiety disorders and neurocognitive impairment (45). Clearly, VR is emerging as a scalable, immersive, and patient-centered therapeutic modality with the potential to transform mental health care delivery.

### 2.3 From evidence to immersion: the *Joviality*™ case example

Building on this growing evidence base, VR has emerged as a promising platform for health education and behavior change, particularly in chronic conditions shaped by modifiable risk factors such as cardiovascular disease, diabetes, and chronic kidney disease. In these conditions, commonly targeted modifiable risk factors include physical inactivity and sedentarism, suboptimal diet, tobacco and alcohol use, poor sleep, and stress-related coping behaviors (46). For example, immersive programs, including virtual grocery tours that teach nutrition principles and simulations that reinforce self-management skills such as blood glucose monitoring, enable patients to practice healthy behaviors within safe and engaging environments (47). Through experiential learning, VR can deepen understanding, strengthen adherence, and potentially reduce healthcare costs (47, 48).

Translating proven behavioral interventions into virtual environments remains a critical challenge, with questions about which principles should guide digital adaptation, curriculum selection, and platform design. *Joviality™* demonstrates a practical bridge between traditional behavioral interventions and next-generation delivery systems: a validated positive psychology curriculum is transferred into VR, maintaining fidelity to its evidence base while leveraging immersion to strengthen engagement and skill practice (7, 8). Developed through collaboration among behavioral scientists, engineers, graphic designers, and clinicians, *Joviality™* transforms a five-module positive psychological intervention into an avatar-guided VR experience delivered chairside during hemodialysis.

Originally developed as a structured, manualized positive psychological intervention curriculum delivered via in-person, mobile, and web delivery (7, 49), the *Joviality™* behavioral intervention was adapted for the Oculus Quest 2 using agile design principles that integrate threedimensional (3D) environments, interactive simulations, and real-time feedback. The *Joviality™* curriculum derives from a structured positive psychological intervention evaluated in randomized trials and feasibility studies among adults with chronic conditions (e.g., diabetes, cancer, HIV) and elevated depressive symptoms (8, 50–52). These studies demonstrated improvements in depressive symptoms, emotional well-being, and stress regulation, mediated in part by increased use of behavioral skills such as gratitude, cognitive reappraisal, and prosocial behavior. The intervention is manualized and skills-based, with clearly defined behavioral components grounded in cognitive and affective science, enabling systematic translation into immersive environments. These components were preserved and delivered through structured, avatar-guided modules that facilitate active skill practice. Participants progress through sequential modules that cultivate core skills, including recognition of positive events, cognitive reappraisal, gratitude, kindness, and mindfulness (see Table 1) (7, 53).

**Table 1:** Curriculum evaluation checklist for VR interventions.

Checklist item	Action step
Does the curriculum align with the target population's demographics and needs?	Evaluate demographic compatibility, openness to technology, and barriers.
Is the curriculum adaptable to a VR platform with interactive 3D environments?	Assess the ability to integrate immersive and interactive elements.
Does the curriculum incorporate activities that actively engage users?	Ensure activities promote hands-on learning and user interaction.
Are the visuals designed to be immersive and customizable for user preferences?	Check for visually appealing and customizable settings.
Is the curriculum modular and organized for intuitive navigation?	Verify intuitive spatial organization and seamless module progression.
Does the curriculum use storytelling to enhance engagement and meaning?	Incorporate narrative elements for deeper user engagement.
Does the curriculum include accessibility features for diverse abilities and needs?	Include features like simple controls, language options, and assistive technologies.
Is the curriculum proven effective for the intended behavioral or health outcomes?	Review evidence of effectiveness for similar populations or settings.
Does the curriculum minimize potential risks, such as motion sickness or emotional distress?	Evaluate and address any risks for the target population.

Across diverse clinical environments, including hemodialysis and lung cancer treatment, *Joviality™* has demonstrated feasibility, safety, and strong user engagement, accompanied by improvements in anxiety, fatigue, and pain (7, 8). These findings underscore the potential of immersive VR technology to translate validated behavioral interventions into scalable, patientcentered experiences, setting the stage for a structured development and evaluation framework. However, to date these results come primarily from single-arm and feasibility studies, and formal head-to-head comparisons with prior non-immersive or traditional formats of the positive psychology intervention are not yet available. A randomized trial comparing *Joviality™* VR with a sham VR condition has recently been completed (7), and future work will extend this agenda to noninferiority and superiority trials directly contrasting immersive and non-immersive delivery.

As this work progresses, positive psychological interventions and immersive tools such as *Joviality™* are entering more diverse clinical settings, and concerns about context, equity, and power in their development and deployment become central. Recent work in positive psychology and intervention science has introduced checklists that prompt consideration of cultural, structural, and ethical dimensions at each stage, including who defines well-being, how contextual vulnerabilities and power dynamics are addressed in clinical encounters, and how benefits and burdens are distributed (54). Our group has similarly emphasized culturally grounded interventions, including qualitative work on how Hispanics/Latinos define well-being (55) and quantitative evaluations of interventions that integrate spirituality and interpersonal connection as core elements (56). The *Transcend Framework* complements these contributions: whereas checklists and culturally focused models guide critical reflection on constructs, sampling, and ethics, *Transcend* focuses on the engineering and design translation of evidence-based behavioral curricula into immersive VR, from curriculum decomposition through asset creation and usability testing, supporting conceptual rigor and practical implementation when adapting behavioral interventions into immersive formats.

## 3 Methods: framework development and implementation process

**Figure 1:**
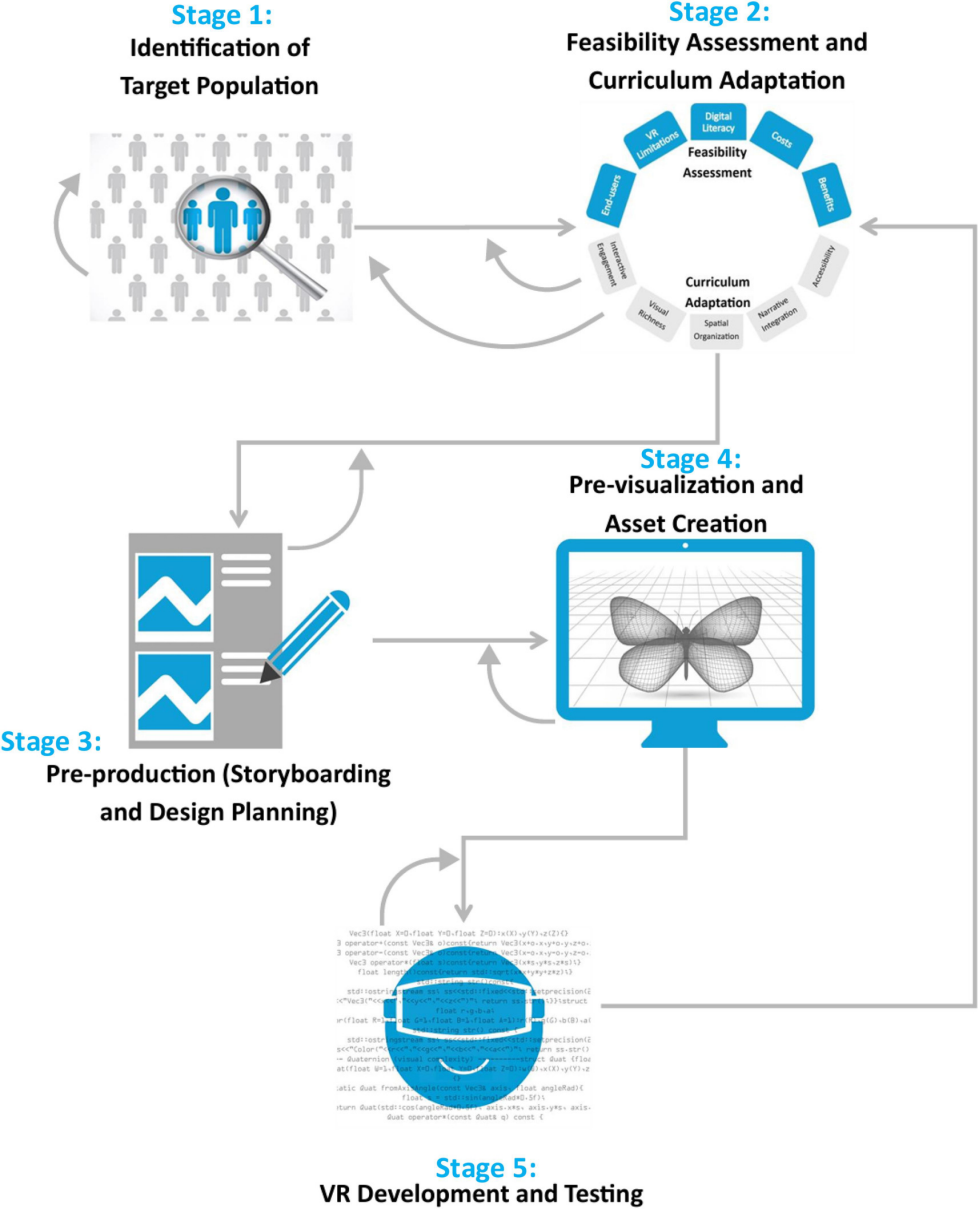
Transcend framework.

Utilizing Agile development methodology (57, 58), and informed by best practices in behavioral science, clinical research, interaction design, game design, and engineering, we developed a novel translational model, the *Transcend Framework* (Translational Engineering of Behavioral Interventions into Immersive Contexts for Engagement and Design). The *Transcend Framework* was created to guide the systematic adaptation of evidence based behavioral interventions into immersive, user-centered virtual environments. It emphasizes iterative design, cross disciplinary collaboration, and end-user engagement to maintain intervention fidelity while fully leveraging the capabilities of VR technology. The framework comprises five primary stages (Figure 1):

- ***Stage 1*—**identification of the target population,
- ***Stage 2*—**feasibility assessment and adaptation of the evidence-based curriculum for VR delivery,
- ***Stage 3*—**pre-production (storyboarding and design planning),
- ***Stage 4*—**previsualization and asset creation, and
- ***Stage 5—***VR development and testing.

In the sections that follow, we describe each stage of the *Transcend Framework* in detail and illustrate its application to the development of Joviality™, transforming a five-module, evidencebased positive psychology curriculum into a fully immersive 3D VR environment. Although developed using a mental health–focused case example, *Transcend* is intended to be domainagnostic and applicable to a wide range of VR applications, including clinical skills training, patient education, rehabilitation, and safety or procedural simulations across home-based, inclinic, and enterprise settings. However, interventions in which intensive motor training and sensorimotor feedback are the primary therapeutic mechanisms demand additional domainspecific engineering and clinical considerations, and therefore fall outside the scope of the illustrative example presented here.

### 3.1 *Stage 1*: assessing population fit for VR delivery

In Stage 1 of the *Transcend Framework*, careful consideration of the target population is essential when transitioning an intervention to VR. The delivery modality must align with the characteristics, needs, and context of the intended end users. Foundational questions guide this decision: *Is VR an appropriate delivery method for the intended behavior change, and is it accessible to the population being served?* Providing VR access in clinical or educational environments already frequented by participants can facilitate engagement and eliminate the financial burden of purchasing equipment. On-site clinical access promotes equity, reduces barriers to participation, and supports consistent exposure within a familiar setting.

Population characteristics are critical determinants of feasibility. Research teams must evaluate whether potential end users have physical or sensory limitations that may restrict VR use (for example, a history of vertigo, seizures, or significant visual impairment) and anticipate potential unintended effects, such as motion sickness or emotional distress (59, 60). For instance, standing on the edge of a tall virtual building may elicit anxiety in someone who fears heights. Openness to digital tools and comfort with new technologies should also be assessed. VR is most appropriate when it offers a clear advantage over traditional methods and when its potential benefits in engagement, therapeutic experience, or clinical outcomes outweigh the associated costs (61). This evaluation should also consider population-specific clinical needs and risks to ensure immersive delivery supports psychological well-being without replacing essential cliniciandelivered care when indicated, such as when suicidality or acute psychological crisis warrants immediate clinical evaluation and care.

In summary, selecting VR should be an intentional, end-user–driven decision. Key questions include: *Does VR enhance the intervention rather than complicate it? Will participants be able to access and use the technology equitably? Does VR improve engagement, outcomes, reach, or cost-effectiveness compared with existing methods? And does the added immersion justify the time, effort, and resources required from both the end-user and the clinical team?* Immersive delivery is particularly appropriate for interventions requiring repeated practice, standardized delivery, or scalable dissemination in settings with limited behavioral health workforce capacity, where digital platforms can extend access without increasing clinician burden.

Beyond feasibility, these questions also raise broader issues of context, equity, and power in immersive intervention delivery. Although *Transcend* is primarily a translational design framework, it operates within clinical environments that are shaped by structural inequities and power asymmetries between patients, clinicians, and technology developers. In line with recent recommendations in positive psychology to foreground contextual vulnerabilities, ethical considerations, and power relations (54), we view Stage 1 as a critical point for examining who is invited into co-design processes, whose goals and values define “success”, and how immersive delivery might unintentionally amplify or mitigate existing inequities (e.g., language exclusion, differential digital literacy, or coercive use of mandated technologies).

### 3.2 *Stage 2*: selecting and preparing an evidence-based curriculum for VR

Stage 2 involves identifying an evidence-based intervention aligned with the target population and intended behavioral outcome. For *Joviality™* VR, the goal was to enhance emotional well-being in adults receiving hemodialysis, a population with high rates of psychological distress. The underlying positive psychological curriculum has been evaluated in randomized trials and feasibility studies across diverse clinical populations, demonstrating feasibility, acceptability, and improvements in emotional well-being and stress management (4951). Prior studies in patients with chronic illness, including kidney disease and other medically intensive conditions, show that these skills reduce depressive symptoms and perceived stress and enhance psychological well-being, outcomes directly relevant to dialysis, where emotional distress is common and independently associated with morbidity, treatment adherence, and quality of life (49, 50, 62, 63).

Positive psychological interventions offer skills-based strategies that strengthen coping, emotion regulation, and resilience, and are particularly well suited to medically ill populations with limited access to specialty mental health care (64). In hemodialysis settings, where prolonged treatment sessions and scarce behavioral health resources create demand for scalable, low-burden supports, these interventions can augment intensive, individual-based clinician-delivered therapy by promoting psychological well-being without disrupting clinical workflows. Immersive delivery further enables standardized, repeatable skill practice during otherwise inactive treatment time, extending behavioral support without increasing clinician workload.

Because the underlying *Joviality*™ curriculum is structured, modular, and skills-based, it was well suited for systematic adaptation to immersive VR. Accordingly, the curriculum was systematically decomposed to separate empirically supported therapeutic components from delivery elements shaped by the original format and clinical context. Core behavioral skills, including gratitude, cognitive reappraisal, savoring, mindfulness, and prosocial activity, were preserved based on prior randomized trials and mechanistic research (7, 49). Delivery features such as session duration, instructional pacing, and interaction structure followed the web-based intervention and were refined using behavioral science expertise, immersive VR safety evidence on cybersickness risk with prolonged exposure, and workflow testing with patient end-users (65). This structured approach preserved validated therapeutic components while enabling systematic adaptation for immersive delivery and aligned established outcome domains, including depressive symptoms, emotional well-being, and stress regulation (7), with the translational principles of the *Transcend Framework*.

Immersive delivery builds on this foundation by extending the intervention's progression from clinician-delivered to self-guided digital formats. *Joviality™* preserves empirically supported behavioral components while augmenting delivery through interactive environments and avatarguided, sequential modules that support experiential skill practice and mastery (7). Headsetderived data on module completion, interaction patterns, and immersion time enable objective monitoring of adherence and exposure while maintaining the program's scope as a self-guided behavioral intervention. In hemodialysis settings, VR is delivered during otherwise idle treatment time, with bilingual narration, hands-free gaze navigation, and clear opt-out options to support accessibility, autonomy, and minimal burden (7). Together, these features illustrate how the *Transcend Framework* guides the adaptation of an evidence-based curriculum into an immersive, self-guided format that remains aligned with clinical workflows and equity-focused design considerations (66).

Building on this foundation, after selecting the curriculum, the study team assessed its suitability for a 3D environment. Five key attributes guided this evaluation: interactive engagement, visual richness, spatial organization, narrative integration, and accessibility (Table 1).

#### 3.2.1 Interactive engagement

The curriculum should support active learning through interaction rather than passive reading or observation. In *Joviality™*, a lesson on positive reappraisal (the practice of reframing a stressful situation to identify constructive or beneficial elements) was delivered through a 360-degree scene at a real bus stop in Urbana-Champaign, where end users virtually “teleported” into the environment, witnessed two actors reacting differently to a similar stressful situation, and interacted with the training module by selecting which actor demonstrated the most adaptive emotional response to the delayed bus. By choosing which reaction was most adaptive, users practiced the behavioral principle in real time within the immersive environment. ***Key Attribute #1:*** *The curriculum must support interactive and immersive scenarios that deepen engagement and promote skill acquisition.*

#### 3.2.2 Designing visually immersive environments

The curriculum should translate into visually compelling environments that sustain engagement and enhance immersion. *Joviality™* applied this principle by incorporating calming and aesthetically rich settings that allowed end users to select their preferred environment. During the mindfulness module, for example, users chose to meditate in a tranquil beach setting or a peaceful garden, while other modules offered views from an outdoor terrace overlooking city or natural landscapes. These visually rich environments reinforced immersion and supported continued engagement. ***Key Attribute #2:*** *Lessons should use visually stimulating and customizable environments to enhance immersion.*

#### 3.2.3 Structuring curriculum for immersive learning

The curriculum must support intuitive spatial arrangement and modular delivery within the 3D environment. Content should be presented in a manner that minimizes cognitive load and guides users naturally through each lesson (67). In *Joviality™*, each module appeared as a panel along a garden wall within the virtual environment.

As users completed one module, the next panel illuminated and became selectable, guiding navigation and reinforcing progress. Modularity also allowed individual lessons to be updated or replaced over time without disrupting the overall experience.

Narrative integration strengthens learning by embedding content within a purposeful storyline. In *Joviality™*, end-users photographed elements of nature in a virtual garden while practicing the skill of noticing positive events. In the following module on savoring, those same images appeared in a three-dimensional gallery where users reflected on, displayed their photos, and made simple photo enhancements, transforming a brief moment of noticing into a sustained positive experience. By carrying tasks forward across modules, the intervention creates a coherent narrative arc that reinforces skill practice and deepens engagement over time. ***Key Attributes #3 & #4****: The curriculum should enable spatial organization, modular design, and narrative integration to support seamless navigation and meaningful engagement.*

#### 3.2.4 Designing for equitable access

The curriculum must be accessible to end users with diverse physical, sensory, and cognitive needs. In *Joviality™*, patients participating during hemodialysis navigated the VR environment hands-free using simple eye gaze and head movements, which eliminated the need for hand controllers while connected to the dialyzer. Visual elements were placed within a forward-facing field of view to accommodate the seated posture and limited mobility common during treatment. The environment was designed so that all interactive objects were reachable through gaze selection, and users could progress through lessons without lifting their arms or adjusting equipment. All modules are designed as seated, stationary experiences/activities in which users do not engage in any virtual locomotion. In addition, text size and contrast were optimized for low vision and ambient lighting variation in treatment bays. Similar considerations, including multiple language options, intuitive interfaces, and compatibility with assistive supports, are essential to ensure that immersive VR remains usable and equitable across population groups. ***Key Attribute #5:*** *The curriculum should prioritize accessibility for diverse abilities, languages, and end-user needs.*

### 3.3 *Stage 3*: pre-production—planning, script development, and storyboarding

*Planning and Preparation*. Effective VR development requires early planning and securing core production resources. Access to a high-fidelity sound studio, motion capture capabilities, video editing software, and a VR development platform ensures technical feasibility and supports a smooth production workflow (Table 2). Preparation also involves a deep understanding of the intended end-users and their needs (57). VR development is inherently multidisciplinary: engineers and designers translate clinical and behavioral content into immersive experiences, while clinicians and behavioral scientists define the therapeutic intent, ensure fidelity to evidence, and safeguard patient safety (58).

**Table 2:** Comprehensive foundational equipment for VR software development.

Equipment	Purpose	Recommended tools
High-Fidelity Audio Recording Studio	Record high-quality audio for voice narration	Zoom Lavalier Mic + Recorder, Adobe Audition, Audio recording booth or sound proofed studio
High-Fidelity Audio Device	Record high-quality audio for immersive soundscapes	Zoom Handheld Audio Recorder, Adobe Audition,
Motion Capture System	Create precise 3D character animations	OptiTrack, Vicon, Rokoko MoCap System
3D scanner	Create precise 3D models from real life objects and environments	LiDAR scanner or a Smartphone with a LiDAR scanner, Photogrammetry apps like Polycam
Video Editing Software	Refine and enhance video content for the VR environment	Adobe Premiere Pro, Final Cut Pro
VR Development Platform	Integrate all elements into a cohesive VR experience	Unity, Unreal Engine
3D Modeling Software	Develop detailed 3D assets for VR environments	Blender, Autodesk Maya
Graphic Design Software	Create textures, icons, and 2D assets for VR interfaces	Adobe Photoshop, Illustrator
High-Performance Computing Workstation	Run resource-intensive development and rendering tasks	VR Ready PCs, Custom-built PCs, NVIDIA RTX GPUs
VR Headsets and Accessories	Test VR applications across different hardware setups	Meta Quest 2, HTC Vive, Valve Index
360-Degree Camera	Capture immersive 360-degree visuals for VR content	Insta360 Pro, GoPro Max
Lighting Equipment	Ensure consistent lighting for video and motion capture	LED Panels, Softboxes
Green Screen and Backdrops	Create clean, chroma-keyable backgrounds for content	Elgato Green Screen, Custom Backdrops
Cloud Storage and Backup Solutions	Safeguard project files and ensure seamless collaboration	Google Drive, Dropbox, AWS
Version Control Software	Manage collaborative coding and asset development	GitHub, GitLab
User Testing Equipment	Conduct usability testing and gather feedback	VR Headset, Eye-tracking equipment, Survey tools
Network Infrastructure	Enable smooth collaboration and online deployment	High-speed internet, Secure VPN

To that end, a detailed end-user profile is developed to guide design decisions and includes age, language, digital literacy, physical limitations, and clinical context. For *Joviality™*, this process informed key design decisions. Members of the product development team conducted on site observations in the hemodialysis clinic to understand treatment workflows, patient positioning, movement constraints, and the sensory environment. Based on these observations, end users navigated the VR environment hands-free with simple eye gaze and head movement to avoid the need for hand controllers while connected to the dialyzer, and the program was developed in both English and Spanish to reflect the linguistic diversity of the patient population. Because dialysis units are frequently noisy due to treatment equipment and clinical activity, audio was mixed at higher baseline volume and optimized for clarity to ensure that spoken instructions remained audible during use. These decisions ensured accessibility, inclusiveness, and alignment with enduser needs.

With a clear understanding of the end user, the team then conceptualizes how each lesson will be visualized and experienced in VR, generating ideas that align with the end-users' linguistic, sensory, and physical abilities, clinical context (e.g., medical settings), and accessibility needs. These concepts are iteratively refined for feasibility and usability, and the finalized elements are incorporated into the Game or Experience Design Document, which serves as the blueprint for VR development.

#### 3.3.1 The VR design blueprint

**Figure 2:**
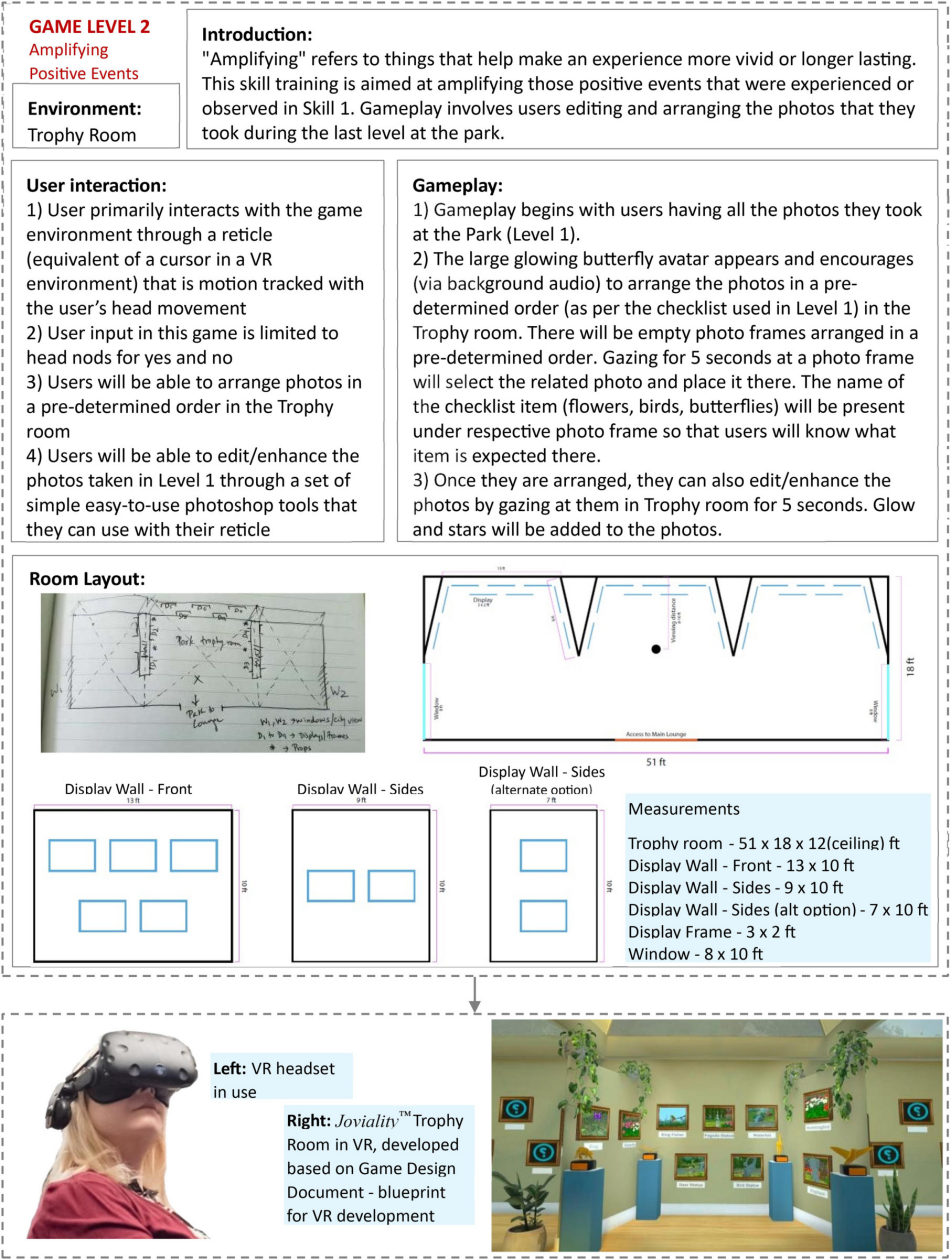
Game design document for the *Joviality*™ amplifying positive events module.

The Game or Experience Design Document serves as the blueprint for VR development (Figure 2). It translates insights from the preparation phase into actionable design specifications and instructions. The document describes each module, its purpose, and the desired end-user interactions. It identifies the VR devices to be used and outlines game mechanics, interaction rules, scene progression, characters, props, and all required visual and audio assets. It also details the characters, environments, and props supported by reference materials such as sample images and design templates to ensure visual consistency throughout development. Critically, this document resolves decisions about interaction (for example, gaze selection vs. hand controllers) based on end-user abilities and clinical context. By specifying visual references, sound design, and animation needs, it ensures coherence in look, feel, and accessibility across modules.

For example, in the mindfulness module of *Joviality™*, the design document specified a living room learning environment with an educational video, ambient fireplace audio, and simple head-guided interaction. The setting was designed to feel familiar and calming, with natural lighting, soft textures, and subtle sound cues that encouraged focus and relaxation. Users then interacted with an art frame to transition to a tranquil beach or garden for meditation. Each environment was rendered with attention to sensory balance to minimize visual overload and promote sustained comfort. Every decision, from prop selection to scene transitions, reinforced ease of use and therapeutic engagement.

#### 3.3.2 Converting scripts to visual storyboards

Storyboarding translates the gameplay script and design document into a series of sequential visual panels that depict how the user will move through each scene. A storyboard functions as a visual blueprint for the intervention, resembling a comic book layout in which each frame illustrates a specific moment or interaction in the VR experience. Each panel includes sketches of the environment, character positions, user actions, and camera perspective, accompanied by written notes on dialogue, timing, lighting, and sound effects. These frames collectively provide a visual timeline that helps the team anticipate how therapeutic content unfolds in three-dimensional space.

**Figure 3:**
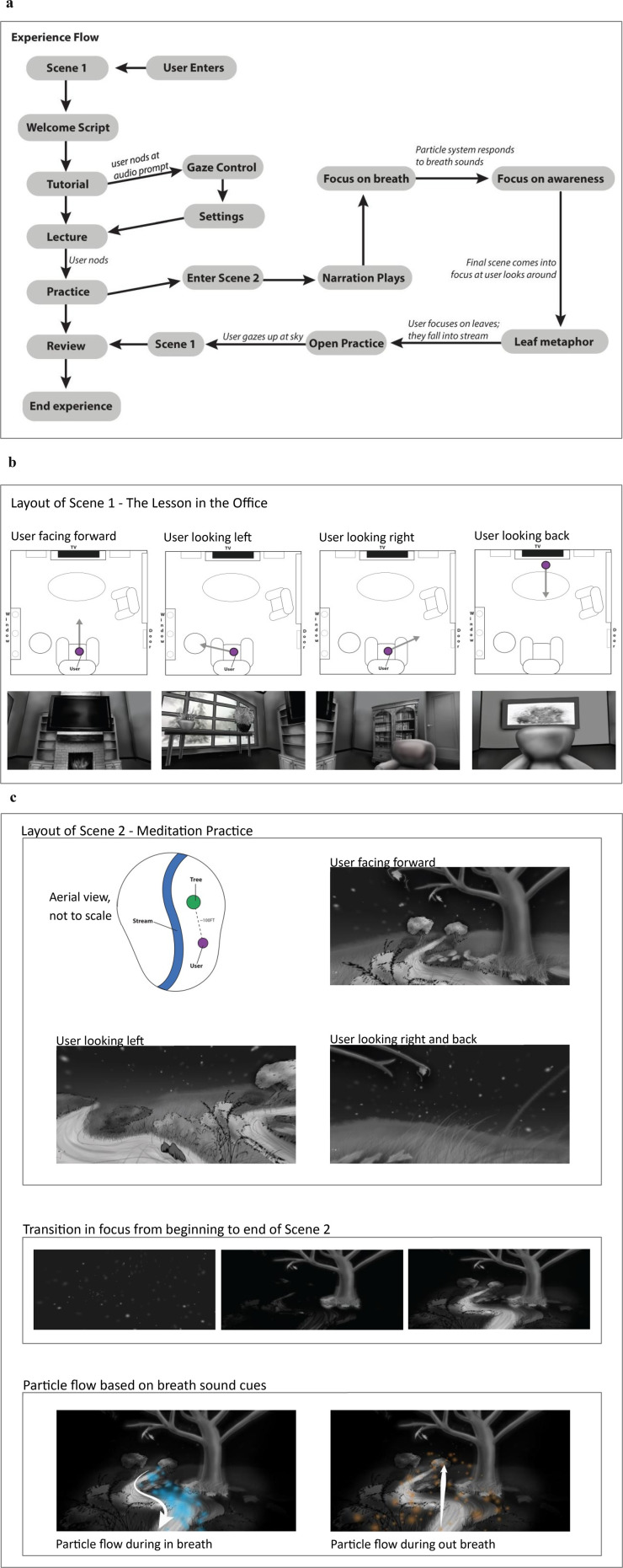
**(a)** experience flow for the *Joviality*™ mindfulness and meditation module. **(b)** Storyboards for Scene 1 of the *Joviality*™ Mindfulness and Meditation Module. **(c)** Storyboards for Scene 2 of the *Joviality*™ Mindfulness and Meditation Module.

Behavioral scientists and clinicians collaborate with designers during this stage to ensure that the emotional tone, pacing, and behavioral skill practice align with the intervention's theoretical framework. Technical annotations specify elements such as field of view, scene transitions, and timing of interactions, allowing developers to replicate the intended experience with precision. Figure 3a shows the experience flow, and Figure 3b and Figure 3c show example storyboards from the *Joviality™* mindfulness module, including the living room layout, placement of furniture and props, meditation transition point, and environmental sound cues.

#### 3.3.3 Applying gamification and sustained engagement

Gamification enhances motivation and sustained engagement, particularly when users vary in age, digital literacy, or physical capacity (68, 69). Interaction pacing and task complexity must align with end users' cognitive load, attention span, and endurance to prevent fatigue or disengagement (70, 71). Behavioral principles such as immediate feedback, goal setting, and reward contingencies can be embedded within the VR experience to strengthen skill acquisition and reinforce positive behaviors over time (72).

Gamification refers to the use of game design elements such as points, progress indicators, challenges, and virtual rewards within a non-game context to increase engagement and persistence (73). These features transform therapeutic exercises into interactive challenges that encourage repeated practice and mastery.

In *Joviality™*, adults undergoing hemodialysis preferred longer interaction windows and slower pacing, while young adult end users in mental health settings responded better to shorter, dynamic interactions with rapid reinforcement. Gamification was applied intentionally to translate behavioral constructs into experiential learning. For example, in the altruism module, end-users virtually selected fruits, vegetables, and shelf-stable foods from a digital basket, placed the items into a box, and mailed it to a virtual family experiencing food insecurity. After sending the package, users interacted with the family through brief visual and auditory cues that conveyed gratitude, reinforcing the link between prosocial behavior and emotional reward. This activity bridged virtual engagement with real-world meaning and provided immediate positive feedback to sustain motivation.

#### 3.3.4 Summary

Pre-production is the foundation of VR development. It requires understanding the end-users, designing interactions that respect their abilities and clinical context, translating curriculum into technical specifications, and visualizing the experience through storyboards before development begins.

### 3.4 *Stage 4*: visual engineering and asset development for immersive environments

As Stage 4 of the *Transcend Framework* begins, building on the comprehensive design document and storyboards, the VR production process transforms two-dimensional visual plans into interactive three-dimensional environments. Storyboards serve as the bridge between conceptual design and software development, guiding how scenes are spatially arranged and how end-users will navigate and interact with them. The production team begins by creating detailed layout maps for each environment, translating storyboard panels into digital blueprints that define spatial organization, user pathways, and points of interaction. Style frames are then developed to establish the visual tone, lighting, and atmosphere of each scene. These frames are reviewed in a 360-degree previsualization format within a VR headset, allowing the team to assess scale, realism, and user perspective before full development begins. For character-driven experiences, designers construct avatars, props, and environmental features that align with the behavioral goals of each module, ensuring coherence and engagement across virtual spaces.

During the 3D production phase, led by the 3D artist and graphic designer, initial prototypes are developed in real time using game engines such as Unity or Unreal to verify appropriate scale and spatial accuracy, particularly for seated or clinical contexts such as hemodialysis. These prototypes are iteratively refined through feedback from the behavioral science and clinical teams to confirm that the environments remain therapeutic, contextually relevant, and easy to navigate. Detailed environments are then developed in modeling software such as Blender or Maya, often integrating real-world scans and textures from 3D libraries to enhance realism. To optimize performance while maintaining fidelity, assets are modeled with low polygon counts and balanced lighting to reduce motion sickness and visual strain. Once finalized, all assets are assembled into cohesive scenes with calibrated lighting, shadow mapping, animation sequences, and environmental effects that mimic real-world conditions.

This stage bridges artistic design with behavioral and clinical insight, transforming abstract concepts into immersive, usable environments that sustain focus and promote emotional engagement. The result is a seamless VR experience that retains both technical precision and therapeutic intent.

### 3.5 *Stage 5*: VR implementation—final development, integration and testing

*Software Development and Integration*. The final phase of VR development focuses on integrating and refining all design components before testing for functionality and end-user experience. Software engineers program interactive elements, animations, and navigation features within the headset environment to ensure seamless performance. Audio and visual components, including voiceovers, character dialogue, ambient soundscapes, and embedded videos, are synchronized and tested for clarity and timing. Post-production adjustments include color grading to achieve the desired visual tone and frame rate optimization to maintain a stable experience of at least 90 frames per second.

#### 3.5.1 Systematic testing prior to deployment

Before large scale implementation, the VR environment undergoes structured testing with multiple participant groups, including members of the development team, the general population, and representatives of the target end-user population. This process verifies technical reliability, confirms intuitive navigation, and ensures that the experience aligns with the intervention's behavioral and clinical objectives.

Testing first occurs within the development team to assess technical reliability and performance. In *Joviality™*, this phase identified mismatches between avatar audio and subtitles, temporary loss of navigation controls that prevented users from advancing through scenes, and missing sound files. Each issue was documented by module and timestamp and returned to the engineering team for correction and retesting. Stress testing confirmed smooth operation and stable frame rates of at least 90 frames per second across headsets and hardware configurations.

Testing with members of the general population provides an independent assessment of usability and accessibility. Participants evaluated the clarity of instructions, ease of gaze-based navigation, and intuitiveness of scene transitions and menu functions. To capture feedback from a wide range of users, the *Joviality™* prototype was also featured at the Illinois State Fair Science Hub, where dozens of attendees interacted with the program. This public setting allowed the team to gather input across diverse demographic and age groups, offering valuable insights into accessibility, comfort, and engagement. Feedback from these sessions and other community participants led to practical refinements, including simplified onboarding screens, larger text captions, clearer button icons, and shortened tutorials to reduce cognitive load for new users.

Target end-user testing ensures the program accommodates the physical, sensory, and cognitive context of the intended population. In *Joviality™*, three hemodialysis patients completed guided sessions during treatment. Several usability issues were identified and resolved. Interactive objects were repositioned within a 180-degree field of view to match the seated posture common in dialysis. In the “I-Spy” module, a virtual bird blended too closely with background foliage, prompting adjustments to contrast and motion cues for better visibility. Patients also noted difficulty hearing over the background noise of dialysis machines, leading to recalibration of voiceover volume and equalization for clarity. Caption size, hitbox sensitivity, and gaze-based controls were also refined to improve comfort and precision. These observations extend beyond this implementation and highlight broader methodological considerations for immersive intervention development in clinical care settings.

Future immersive intervention studies should treat clinic-specific engineering constraints as core design requirements rather than downstream implementation details. In acoustically challenging treatment environments, investigators should predefine intelligibility targets for spoken content, test performance under routine clinical conditions, and offer redundant visual channels so that key information remains understandable when audio is compromised. Caution is warranted with noise-canceling audio in medical settings, where awareness of clinician communication is essential if urgent safety issues arise; design choices should therefore preserve situational awareness through transparency features, single-ear delivery, or alternative audio strategies when appropriate (74, 75).

For end users with motor or sensory limitations, interaction modalities should be explicitly specified (for example, gaze-based input, dwell timing, head pose, or controller use), and accessibility thresholds such as caption legibility, interaction tolerance, and field-of-view placement should be evaluated against predefined usability criteria. As immersive technologies evolve, systems should incorporate multimodal accessibility features, including audio, haptic, and adaptive visual supports, to enable use by individuals with visual or auditory impairments. These parameters should be prespecified and prospectively assessed to ensure accessibility, safety, and usability in real-world clinical settings (76, 77). Consistent with these principles, findings from all testing rounds were integrated into successive software builds until defect logs were cleared and usability benchmarks were met, and the resulting *Joviality™* prototype operated reliably, demonstrated intuitive usability, and was ready for larger-scale feasibility evaluation.

## 4 State of the art and lessons learned

The development of *Joviality™* demonstrates how advances in VR can extend validated behavioral interventions into immersive, user-centered environments. State of the art VR design now merges behavioral science, clinical expertise, and engineering to create interventions that are both evidence-based and technically precise. Central to this process is explicit identification and preservation of empirically supported therapeutic components, ensuring that immersive adaptation maintains fidelity to the intervention's evidence base while allowing delivery optimization (78, 79). Behavioral scientists establish therapeutic intent and fidelity, clinicians delineate therapeutic goals as well as usability and safety requirements, and designers and engineers translate these specifications into responsive virtual environments.

This multidisciplinary process represents a shift from viewing VR as an adjunct technology to recognizing it as a full implementation platform capable of delivering theory driven interventions at scale. This approach is particularly appropriate for interventions requiring standardized delivery, repeated skill practice, or scalable dissemination in resource-constrained clinical environments, where immersive platforms can extend behavioral support without increasing clinician burden and improve long-term delivery efficiency despite higher initial development costs (80). To systematically guide such translations and ensure preservation of therapeutic fidelity, our team draws on the five-stage *Transcend Framework*, a structured model that integrates behavioral theory, clinical insight, and iterative engineering to ensure that interventions remain both scientifically rigorous and technologically feasible (81). While *Joviality™* illustrates a mental health application, the same staged process is suitable for other VR use cases, particularly structured, curriculum-based interventions for clinicians, patients, and trainees delivered in private, clinic-based, or supervised institutional settings.

Widespread implementation of the *Transcend Framework* will also require deliberate attention to practical barriers. Effective translation of behavioral interventions into immersive VR typically depends on multidisciplinary teams that bring together behavioral scientists, clinicians, designers, and experienced developers, resources that may be unevenly distributed across institutions. In the case of *Joviality™*, collaboration across multiple academic units, together with access to a dedicated VR laboratory within the university's Center for Innovation in Teaching and Learning (CITL), which houses advanced VR hardware and is closely integrated with one of the nation's leading engineering programs, made this type of development feasible. Moreover, because VR hardware, software, and interaction paradigms continue to evolve, the framework should be treated as a flexible guide that can be periodically updated so that future applications remain aligned with emerging technologies and best practices. Consistent with this principle, the current implementation of *Joviality™* is delivered on current-generation VR headsets but was developed in Unity using modular, headset-agnostic architecture to accommodate successive generations of VR hardware and to support future extension to XR platforms that leverage advanced eye-tracking, haptic interfaces, and biometric sensing. In this way, both the framework and its concrete implementations can evolve in step with advances in immersive technology.

Several key lessons emerged from the *Joviality™* initiative. Early engagement with endusers is essential to identify physical, cognitive, and sensory factors that shape design decisions. Iterative testing across diverse participant groups helps ensures that VR environments are not only technically robust but also behaviorally meaningful and equitable. Collaboration between clinical and technical teams must balance immersion with simplicity, while cultural adaptation strengthens relevance and sustained engagement. Personalization of VR experiences can be achieved by tailoring both therapeutic content and the immersive setting. For example, narrative content, difficulty, feedback, and goal-setting can reflect individual profiles of modifiable risk factors (e.g., disease stage, activity level, dietary habits, stress triggers), while environmental elements such as preferred meditation settings (e.g., a garden vs. a beach in *Joviality™*) are adjusted to user preferences; together, these strategies align game or experience design with principles of personalized behavioral medicine (82).

These lessons also underscore the importance of explicitly considering mode (e.g., selfguided, facilitated, or blended) and format (e.g., individual vs. group-based) when applying *Transcend*, as these choices can shape engagement and clinical outcomes in mental health interventions (83). For mode, evaluation should include the dose (e.g., frequency and duration) and the type of support (e.g., self-guided and clinician contact in facilitated or blended model) (84) because greater human support in internet-based and digital health settings is often associated with higher adherence (85). Furthermore, when it comes to format, evaluation should include feasibility and retention patterns; when group-based delivery is used, group process indicators (e.g., attendance, interaction quality/cohesion, and facilitator fidelity) should also be assessed (86). Collectively, these insights highlight that successful VR interventions depend as much on behavioral design and user input as on software sophistication.

Frameworks such as the checklist proposed by Gaffaney and Donaldson (54) emphasize contextual vulnerabilities, ethical considerations, and power dynamics across all phases of intervention research. We intend for *Transcend Framework* to be used alongside such guidance, for example by applying equity- and power-focused criteria when assessing population fit (Stage 1), selecting curricula and outcome measures (Stage 2), and making design choices about representation, voice, and autonomy in immersive environments (Stages 3–5). Integrating these approaches strengthens *Transcend's* conceptual grounding by pairing detailed translational design steps with a systematic focus on context and power in clinical settings. Attention to cultural and structural diversity is central to our own work, which applies a well-being lens that resists a universal, “one-size-fits-all” model and instead situates intervention design within the ecological contexts, values, and lived experiences of the populations served (55, 87). For behavioral scientists, the experience with *Joviality™* underscores that developing for VR involves more than adapting existing interventions to a new format. It requires reimagining how users interact with content, process feedback, and sustain behavior change in an immersive context. The next frontier lies in merging behavioral theory with human computer interaction, accessibility, and iterative testing to ensure interventions remain both impactful and equitable. Future work will explicitly compare immersive and non-immersive versions of the intervention, using quantitative and qualitative outcomes (e.g., engagement, emotional well-being, usability) to evaluate whether VR delivery provides added benefit beyond established formats and to support formal non-inferiority testing when appropriate. More broadly, *Joviality™* represents only a single case example of the *Transcend Framework* in action, focused on a structured positive psychology curriculum in hemodialysis and oncology settings and demonstrating feasibility, usability, safety, and improvements in anxiety, fatigue, and pain among adults with lung cancer and other serious illness. Larger-scale, multi-intervention evaluations across diverse behavioral targets and clinical environments, including behavioral and health outcomes, are an important next step to determine whether the *Transcend Framework* supports effective translation of other evidence-based therapies into immersive VR and to establish its generalizability and utility. Behavioral scientists are uniquely positioned to guide this evolution, ensuring that VR continues to advance the science of behavior change while expanding access to those who benefit most.

## 5 Conclusion

VR has evolved far beyond its origins in entertainment to become a transformative tool across disciplines, particularly in healthcare. Advances in computing power, high-resolution graphics, and intuitive interface design now allow clinicians and patients to engage in immersive environments that promote learning, skill acquisition, and emotional well-being. Once limited by cost and accessibility, the steady decline in headset prices and improvements in usability have made VR increasingly viable for both clinical and community settings. As behavioral scientists adopt VR as a delivery platform, it is essential to integrate principles of health equity and inclusivity so that the benefits of these technologies extend across race, ethnicity, socioeconomic status, and levels of technical proficiency.

The development and implementation of *Joviality™*, a VR based positive psychological intervention, illustrate both the opportunities and challenges inherent in translating evidence-based curricula into immersive platforms (7, 8, 88). Using a systematic, user-centered approach, the project moved through each phase of development, from assessing target populations to iterative testing, to ensure accessibility, engagement, and functionality. Key design choices, such as implementing head-controlled (hands-free) navigation for patients receiving hemodialysis and adjusting visual and auditory elements based on pilot feedback, demonstrate how behavioral content can be optimized for therapeutic environments. The shift from a text based program to a hands-free, avatar guided experience addressed critical barriers faced by hemodialysis patients, including limited hand mobility, difficulty navigating web based interfaces, and literacy demands associated with written content (53). These refinements show how VR can bridge the gap between traditional behavioral interventions and next generation delivery systems, improving access and adherence among populations with complex medical needs (8).

Our prior studies among culturally diverse groups, including Hispanic/Latino adults and Korean adults, further show the promise of culturally adaptable VR interventions (55, 89). Enduser testing with older Korean immigrants emphasized the importance of cultural relevance, emotional resonance, and usability in the Korean language version of *Joviality™*. Participants identified several strengths of the immersive approach, such as cultivating appreciation for nature to evoke calm and accomplishment, fostering gratitude through interactive reflective exercises, and increasing engagement and comprehension through gamified module design. Many participants expressed interest in continuing these activities at home using VR devices, suggesting the potential for sustained self-practice and long-term behavioral maintenance.

Looking ahead, VR is poised to play an expanding role in health education and behavior modification, particularly for conditions driven by modifiable risk factors. As demonstrated through the *Joviality™* development process, iterative design and comprehensive testing are essential to ensure interventions are functional, user-friendly, and impactful. While final software deployment marks a technical milestone, ongoing adaptation remains necessary to accommodate emerging technologies and evolving user needs. Future directions include expanding access among hesitant users by highlighting practical benefits, integrating artificial intelligence to personalize avatars and environments in support of precision behavioral medicine, and embedding multilingual capabilities to reach linguistically diverse populations.

Across these stages, the *Transcend Framework* provides the organizing structure that links design decisions to behavioral theory, clinical context, and engineering processes. It clarifies how interventions progress from conceptualization to deployment, ensuring that immersive translation remains systematic, theory-driven, and responsive to end-user needs. In this way, *Transcend* provided the roadmap that guided the development of *Joviality™* and now offers a replicable model for future VR-based behavioral interventions. The lessons derived from *Joviality™* offer a pragmatic framework for behavioral scientists and developers seeking to use VR to enhance health, engagement, and equity across clinical and community settings.

## Data Availability

The original contributions presented in the study are included in the article/Supplementary Material, further inquiries can be directed to the corresponding author.
